# Community interpreting in Germany: results of a nationwide cross-sectional study among interpreters

**DOI:** 10.1186/s12889-024-18988-8

**Published:** 2024-06-11

**Authors:** Saskia Hanft-Robert, Mike Mösko

**Affiliations:** 1 Department of Medical Psychology, University Medical Center Hamburg-Eppendorf, Martinistraße 52, 20246, 0049 40, 7410 56684 Hamburg, Germany; 2 Department of Applied Human Sciences, University of Applied Sciences Magdeburg-Stendal, Stendal, Germany

**Keywords:** Community interpreting, Public service interpreting, Qualification, Working conditions, Psychosocial distress

## Abstract

**Background:**

Community interpreters (CIPs) play a crucial role in various community services, including healthcare, when service providers and users do not share a common language. However, there is a lack of evidence-based data on this population globally. This explorative cross-sectional study aims to gain a better understanding of CIPs and their work in Germany.

**Methods:**

A nationwide online survey was conducted among CIPs in Germany to collect data on their qualification background, working conditions, mental health, interpreting-related psychosocial distress and sociodemographics. Participants were recruited through interpreting pools, training institutions and migrant organizations. Data were analyzed descriptively, dependent t-test, multiple logistic and hierarchical stepwise regression analyses were performed to predict participation in interpreting-specific training, interpreting competence and interpreting-related psychosocial distress.

**Results:**

Across all 16 federal states, *N* = 873 responses were used for analysis. Most participants are female (74%), born abroad (77%) and have a high level of education (69%). The vast majority interpret occasionally in their leisure time (44%) and are self-employed/freelance (51%). 34% interpret solely or additional on a voluntary basis (unpaid). The median hours of interpreting per month are 10 h, 75% do not exceed 30 h. On average interpreters work in four different settings. 69% attended any kind of interpreting training with a median of 25 h in total. Interpreting in more settings emerged as an associated factor with participation in training. Of those who have never attended any training, 69% consider themselves as rather/very competent in interpreting. Interpreting more frequently, having less severe anxiety symptoms, getting higher and more often paid and being less satisfied with the payment is associated with self-reported interpreting competence. In total, 36% reported moderate or severe psychosocial distress regarding interpreting. Higher general psychosocial distress and depressive symptoms, higher interpreting frequency and lower payment satisfaction were found to be associated with higher distress regarding interpreting. Additionally, factors such as precarious work conditions, lack of recognition and discrimination (e.g. racism and sexism) were reported as distressing.

**Conclusion:**

This study provides a first comprehensive evidence-based national database on CIPs in Germany. The findings can be valuable for the development of qualifications, guidelines, policies and the process of professionalizing the field of CIPs.

**Supplementary Information:**

The online version contains supplementary material available at 10.1186/s12889-024-18988-8.

## Introduction

### Growing linguistic diversity and the need for community interpreters

Ongoing globalization and migration are leading to increased cultural and linguistic diversity of populations worldwide [1, 2]. However, the growing cultural and linguistic diversity is often not reflected among public service providers or in community settings, such as health care [3, 4]. The severe consequences and risks associated with linguistic discordance between service users and providers, such as miscommunication and, particularly in healthcare settings, misdiagnosis, ineffective treatment and lower treatment satisfaction, have been demonstrated in several studies [5–12], which emphasizes the importance of language-sensitive services. When the service provider and user do not share a common language, the use of community interpreters (CIPs) can be one approach to facilitate effective communication, in addition to nonverbal communication, receptive multilingualism, technological translation tools and family members or multilingual personnel as interpreters [12–20].

Although Community Interpreting (CI) can be considered the oldest form of interpreting, it established itself as a field of research at the beginning of the 1990s and is thus a relatively young research topic [21–23]. In the international literature, terms such as public service, liaison, dialogue, or cultural interpreting as well as more setting-specific terms such as medical or legal interpreting are commonly used when discussing CI, demonstrating the lack of consistent terminology [21, 24–27]. The terminological ambiguity stems from the fact that most countries, such as Germany, do not have a legally protected professional title for people working as interpreters in community settings. In this study, the term CI was chosen because it is used by the International Organization for Standardization (ISO) in their Guidelines for CI (ISO 13611:2024) [28]. CI is often defined in distinction from other types of interpreting, such as conference interpreting, in that it takes place within the community to facilitate equal access for community service users when they do not share a common language with the service provider [21–23, 25, 29–31]. Community Interpreters (CPIs) usually work in a dialogic encounter between two, sometimes more, speakers either on-site or via telephone or video remote link. Most often, the focus is on bilateral interpretation (CIPs transferring utterances into both working languages) with an emphasis on the consecutive mode (translation once the speaker stops speaking) [23, 25, 27, 29]. In many countries, there is no legal entitlement to the provision of interpreting services in most community settings, resulting in a lack of formal standards regarding CIs’ qualifications, organization and funding.

### Qualification of community interpreters

In the public perception, CI often receives little recognition and tends to be seen as a charity or volunteer activity provided by bilinguals as an act of social support, rather than a professional service, resulting in a lack of legal regulation regarding CIPs’ formal qualification [25, 32]. However, over the past decades, CI’s recognition has evolved in some countries to a profession that requires qualifications, including specific training and certification, as well as legal and ethical regulations [30, 32]. Despite this evolution, many countries lack legally anchored standards regarding CIPs’ formal qualification, resulting in a great diversity of training institutions and programs as well as official organizations that certify CIPs [23, 29, 33–37]. The absence of standardized requirements allows anyone to work as a CIP based primarily on the subjective self-assessment of their skills rather than their actual qualification [25]. Australia stands out as an exception, placing a strong emphasis on adhering to legal standards and guidelines in CI. The National Accreditation Authority for Translators and Interpreters (NAATI) serves as the authoritative body for certifying translators and interpreters nationwide, with NAATI certification often being a prerequisite for interpreters working in community settings [38]. Recognizing the need for globally applicable standards, the International Organization for Standardization (ISO) developed the Guidelines for Community Interpreting first in 2014 [28]. It addresses CI as a profession characterized by specific competencies, qualifications and ethical principles, and not as an informal practice that can be provided by multilingual laypersons such as family members. By offering international recommendations, fundamental principles, and best practices, the ISO guidelines aim to ensure the quality of CI services [28].

Consistent governmental regulations and frameworks in terms of CIPs’ qualifications are also lacking in Germany [39, 40]. Only court interpreting could be considered an exception in Germany, as it is the only setting in which swearing-in is usually required. However, the criteria to be met to be sworn-in vary among the federal states, formal qualification is not always needed, and ad hoc swearing-in without examining the interpreter’s qualifications still occurs [39]. In recent decades, numerous training and certification programs have also emerged in Germany. However, these programs differ significantly in terms of content, scope, teaching approach, duration, and certification. This lack of standardization has contributed to an even less regulated and more confusing CI market. As a result, CIPs’ level of qualification spans a wide spectrum, ranging from no training at all to a few hours of interpreting-specific training to a formal university degree in interpreting [40]. In Switzerland, the Swiss Association for Intercultural Interpreting and Mediation (INTERPRET) reported that out of 3,122 interpreters among 18 interpreting pools, 12% had no interpreting-specific qualification and an additional 33% were currently in training in 2022 [41]. In Germany, a recent study conducted by Geiling et al. (2022) showed that among interpreters working in refugee care (e.g., (mental) health care, authorities, psychosocial counseling or police), 15% have an interpreting degree from a university or college, and 67% have any kind of training for interpreting for refugees [42]. However, there is still a scarcity of comprehensive evidence-based and national data regarding the qualifications of CIPs in Germany. Moreover, in the absence of professional standards in many community settings that determine who can officially practice as a CIP, the composition of the CIP population remains largely unknown.

### Organization and funding of community interpreting services

In most countries, a lack of legally established standards also exists regarding the organization and funding of CIPs, leading to CI services usually being organized and funded on a nongovernmental level [34, 35, 39]. Additionally, it must be acknowledged that the provision and funding of (qualified) interpreting services always depend on the context and resources of the respective country. For instance, in some countries like South Africa [16], formal interpreting services and specifically trained CIPs are rarely available, and CI usually occurs informally and on a voluntary basis [43, 44].

In Germany, there is no legal entitlement to interpretation services, with a few exceptions such as sign-language interpretation or criminal proceedings at court [39]. Consequently, there is no formal legal framework governing the funding or organization of CI services [39]. CI services in Germany are mostly organized on a local level, used on a voluntary basis and funded through nongovernmental organizations or by the individuals who hire CIPs to facilitate communication, e.g., service providers or service users [39]. However, the lack of evidence-based data makes it difficult to comprehensively assess the organization and funding of CIPs in Germany and across countries.

### Community interpreters’ working conditions and psychosocial distress

CIPs often work with vulnerable populations in emotionally intense and pressured situations, such as hospitals, legal proceedings, or social services, where they play a crucial role in facilitating communication between service providers and users [25]. Thus, some attention has been drawn to the psychological impact of interpreting in community settings and it is emphasized that not only the content (e.g., interpreting traumatic experiences) but also the poor working conditions (e.g., lack of training, supervision and preparation, time pressure, low payment, unregulated labor market) and the interaction with the interlocutors (e.g., conflicting expectations, discrimination, lack of recognition, identification with the service user) could be emotionally distressing for CIPs [45–49]. However, there is a paucity of evidence-based data across countries concerning CIPs’ working conditions and the psychosocial distress associated with interpreting. The few studies available tend to focus on specific migrant groups or interpreting settings. For instance, Geiling et al. (2022) found increased psychological distress among CIPs working in refugee care in Germany. In addition, dissatisfaction with payment and more traumatic content were linked with work-related exhaustion in their study [46].

## Aim of the study

The purpose of this epidemiological study was to shed light on the work of CIPs in Germany. While most research on CI addresses one specific setting, e.g. medical, legal, or social service settings [50] or a specific migrant group, e.g. refugees [42], we did not focus on a particular setting or group of clients since CIPs most often work in more than one setting with diverse migrant groups [51]. We aimed to.

1. collect data to make a start on describing CIP’s sociodemographic profile, working conditions, (formal) qualification background, mental health status, and psychological distress regarding interpreting;
2. investigate factors that differentiate between people who have taken part in interpreting specific training or not;
3. identify factors associated with self-reported interpreting competence;
4. identify factors associated with psychological distress regarding interpreting;
5. explore differences between interpreting settings of CIPs in terms of sociodemographic and work-related variables;

## Materials and methods

The study was conducted at the Department of Medical Psychology at the University Medical Centre, Hamburg-Eppendorf, in cooperation with the Federal Association of Nonstatutory Welfare and the Federal Chamber of Psychotherapists. A national cross-sectional study design was applied.

### Development of the questionnaire

Due to missing similar studies, the questionnaire was developed by the authors in a multistage process. (A) Based on the literature review, relevant themes were selected. (B) These themes were initially discussed and supplemented in an interdisciplinary workshop with four experts from the fields of interpreting and translation studies, linguistics, and psychology. (C) A second workshop with twelve experts working in different CI pools, agencies, and training institutions across Germany was conducted online to discuss the selected themes as well as potential recruitment strategies. (D) The questionnaire was subsequently expert-validated by six scientists from the fields of translation and interpreting studies, linguistics and psychology. (E) Finally, cognitive interviews were conducted with five CIPs to examine whether participants’ interpretations were consistent with the intended meanings of items. The probing approach with a mix of proactive and reactive probes was chosen [52]. The cognitive interviews were also used to evaluate the questionnaires’ comprehensibility and feasibility with people from the target group. Based on their feedback, some questions were rephrased and linguistically simplified. The final questionnaire consisted of 48 items, divided into four sections:

#### 1) Sociodemographic variables

The survey included single- or multiple-choice questions on gender, age, migration (including reasons for migration, citizenship, country of origin, and length of stay in Germany in years), educational level, recognition of highest educational qualifications in Germany, employment in addition to interpreting and participants’ federal state.

#### 2) Work-related variables

Single- or multiple-choice and open questions were developed to assess work experience as a CIP in years, frequency of interpreting per month in hours, type of employment as a CIP (freelancer, employed, both), interpreting settings (healthcare in general, psychotherapy, social service, authority, education, legal service, court, police, other), type of interpreting (on-site, telephone, video), frequency of payment (5-point Likert scale ranging from “1 = never” to “5 = always”), amount of payment in Euros, satisfaction with payment (5-point Likert scale ranging from “1 = very low” to “5 = very high”), working languages and availability and need of interpreting-related support services.

#### 3) Qualification

Hours of training, topics covered in training, and passed exams were assessed with single- or multiple-choice questions. Additionally, specific structural and personal barriers to attending training were assessed on a 5-point rating scale ranging from “1 = Does not apply at all” to “5 = Fully applies”. An open question was included to capture other factors that prevent participation in training. Subjective training need was also assessed on a 5-point rating scale ranging from “1 = Not at all needed” to “5 = Highly needed”. Similarly, self-reported interpreting competence was assessed on a 5-point rating scale ranging from “1 = Not competent at all” to “5 = Very competent”.

#### 4.1) Psychological distress

The German version of the NCCN Distress Thermometer was used to assess general psychological distress [53]. The easy-to-use screening tool was originally developed by the National Comprehensive Cancer Network (NCCN) to assess psychosocial distress in oncology patients [54]. To date, it has also been used in several other settings due to its high acceptance, brevity, and practice orientation [55]. The NCCN Distress Thermometer is an 11-point Likert scale ranging from “0 = no distress” to “10 = extreme distress”. Participants indicate their levels of distress over the course of one week before the assessment. A cutoff of 4 + is recommended to identify moderate to severe levels of distress [56]. A meta-analysis found a pooled sensitivity of 81% and a pooled specificity of 72% [57].

The NCCN Distress Thermometer was slightly adapted to measure psychological distress regarding interpreting: “Please indicate the number (0–10) that best describes how stressful you currently experience interpreting.”

#### 4.2) Mental health outcomes

CIPs’ mental health was measured as depressive symptoms with the German Patient Health Questionnaire-2 (PHQ-2) [58] and anxiety with the German General Anxiety Disorder-2 (GAD-2) [59]. Both instruments are part of the Patient Health Questionnaire-4 (PHQ-4) [60].

The PHQ-2 is a validated screening instrument for depression [58, 61]. On a two-item scale, participants had to rank the frequency of symptoms (little interest or pleasure in doing things; feeling down, depressed, or hopeless) in the last two weeks on a 4-point Likert scale ranging from “0 = not at all” and “3 = nearly every day.” The sum score (range 0 to 6) indicates the degree of depression, whereby a higher score suggests stronger depressive symptoms (0–2 = “low depressive symptoms”, scores of 3 and above = “high depressive symptoms”) [61]. With a sensitivity of 79% and a specificity of 86% for any depressive disorder, the scale shows adequate psychometric properties [58]. The Cronbach’s alpha reliability score, which calculates the internal consistency of a test or scale [62], was 0.66 for the present sample. While this score is slightly below the conventional threshold of 0.70, it can still be considered acceptable [63, 64].

The GAD-2 is a two-item screening instrument for general anxiety, consisting of the two psychometrically best items from the GAD-7 [59]. Participants had to rank the frequency of symptoms (feeling nervous, anxious or on edge; not being able to stop or control worrying) in the last two weeks. Each item score ranges from “0 = not at all” to “3 = nearly every day”, resulting in a sum score between 0 and 6. A higher score suggests stronger general anxiety symptoms, with a cutoff of 3+ [59, 65]. A systematic review and diagnostic meta-analysis showed adequate psychometric properties, with a pooled sensitivity of 76% and a specificity of 81% for identifying any anxiety disorder [65]. The Cronbach’s alpha reliability score for the present sample was 0.68.

### Participants and data collection

Any person aged 18 years or older working as a CIP in community settings in Germany could participate in the study. No further inclusion or exclusion criteria were defined. In preparation for conducting logistic regression analyses, the required sample size was calculated using G*Power Version 3.1. To reliably demonstrate Odds Ratios of 1.5 and above, a total sample size of 308 participants was needed.

Since CIPs are not organized in a formal way in Germany, a convenience snowball sampling approach was used by reaching out to institutions that had access to the target population. Based on an online search, 112 interpreting pools, agencies, and training institutions for interpreters (including the ones that participated in the second workshop of the questionnaire development) across all federal states as well as relevant nationwide organizations were contacted directly via email explaining the purpose of the study and asking them to forward the study invitation to their affiliated CIPs. The survey was also sent to various social and healthcare institutions that work with CIPs on a regular basis. Following the snowball sampling approach, participants were asked to share the study link with other CIPs at the end of the survey. Of the first 250 participants who provided an email address at the end of the survey, every second participant received a 20 Euro voucher from wunschgutschein.de. The costs for the vouchers were fully covered by the Department of Medical Psychology at the University Medical Center Hamburg-Eppendorf. Data collection was conducted from June to August 2022 using the online survey tool LimeSurvey (Version 2.62.2 + 170,203).

### Data analysis

In a first step and to answer our first research objective (describe CIP’s sociodemographic profile, working conditions, (formal) qualification background, mental health status, and psychological distress regarding interpreting), we computed descriptive statistics and reported means (*M*), standard deviations (SD), frequencies (n), and percentages (%). Concerning our second research objective (investigate factors that differentiate between people who have taken part in interpreting specific training or not), we employed dependent t-tests to examine differences between participants who have participated in training and those who have not. Subsequently, we conducted logistic regression analyses to identify variables contributing to the likelihood of being trained specifically for interpreting. Due to the exploratory nature of the study, we included variables that revealed significant differences in the t-test in the regression analyses.

For the third (identify factors associated with self-reported interpreting competence) and fourth objectives (identify factors associated with psychological distress regarding interpreting), we used Spearman’s correlation analyses and multiple hierarchical stepwise regression analyses. Variables were included in the regression analyses as independent variables if they correlated significantly with the outcome variable in the correlation analyses.

To investigate the fifth objective (explore differences between interpreting settings of CIPs in terms of sociodemographic and work-related variables) crosstabs as well as multiple logistic regression analyses on various outcome variables, using the interpreting setting variables as predictors, and a stepwise forward selection procedure for variables were performed. To this end, the following variables were dichotomized into “0” and “1”: Age, gender, education, interpreting experience, frequency of interpreting, hours of training, interpreting competence, frequency of payment, amount of payment, and appropriateness of payment. All metric variables were dichotomized using the median split. For the variables education, frequency of payment, appropriateness of payment and interpreting competence, dummy variables were computed with “low education” = 0, “never payment” = 0, “payment is very or rather low” = 0 and “no competence” = 0. Complete case analyses were conducted, and participants with any missing data were excluded. No further analyses regarding missing data were conducted. All analyses were performed using IBM SPSS Statistics Version 28.0.1.1 [14]. and R Studio Version 2021.09.0.

### Ethical considerations

We obtained Ethical approval in writing from the Ethics Committee of the University Medical Centre Hamburg-Eppendorf (29 July 2021; LPEK-0360). At the beginning of the survey, we informed participants about the aim and procedure of the online survey; the chance to win a voucher after completing the questionnaire; and that study participation is voluntary and data collection anonymous. A detailed study and data protection information sheet could be downloaded via an external link. Before starting the questionnaire, participants had to give their consent for the data to be used for the purpose of this study.

## Results

In total, 1,199 people clicked on the online survey and 897 answered at least one question. We excluded participants with several nonlogical answers (e.g. country of origin “Klingon”) or repeated participation (*n* = 6) from the analysis. Since sign language interpreters differ from the group of CIPs in terms of legal regulations, qualifications and working conditions, we excluded them (*n* = 18) from this study. Finally, we analyzed data from 873 participants across all 16 federal states in Germany, with most participants residing in Baden-Württemberg (28.4%), Bavaria (15.3%) and North Rhine-Westphalia (9%). The least represented federal states were Brandenburg (0.1%) and Saxony-Anhalt (0.6%).

### Descriptive statistics

#### Sociodemographic variables

##### Age, gender, education level, occupational status

The majority of the sample is female (74.2%) and aged from 18 to 88 years (*M =* 43.84, *SD =* 12.77). Following the International Standard Classification of Education (ISCED) [66], the vast majority reported a high level of education (69.1%). In addition to interpreting, most of the participants are employed part-time (25%), full-time (20.1%) or were self-employed/freelancers (18.1%).

##### Migration history

A total of 77.1% were born abroad, with Russia (9.9%), Syria (9.5%) and Ukraine (9.5%) being the most common countries of origin. The main reasons for migration are family reunion (39.2%) and political, legal or humanitarian reasons (28.2%). Those who immigrated are residing in Germany for *M =* 21.51 years on average (*SD =* 13.12). All sociodemographic variables are summarized in Table 1.

**Table 1:** Sociodemographic characteristics of the sample (*N* = 873)

	*n*	value
**Gender**	**708**	
Female	525	74.2%
Male	163	23%
Other	6	0.9%
Not specified	14	2%
*Missing value*	*165*	-
**Age (in years)**	**706**	MED = 43 years (IQR 34–53) MW = 43.84 years (SD = 12.77, range = 18–88)
*Missing value*	*167*	-
**Own migration history**	**708**	
Born abroad (1. Generation)	546	77.1%
Born in Germany	162	22.9%
*Missing value*	*165*	-
**Migration history of parents (if born in Germany)**	**162**	
Both parents born in Germany	74	45.7%
Both parents born abroad (2. Generation)	60	37%
One parent born abroad (2. Generation)	28	17.3%
*Missing value*	*0*	-
**Reasons for migration (if born abroad)**	**505**	
Family reunion	197	39.2%
Political, legal or humanitarian	142	28.2%
Education	126	25%
Work	40	7.6%
*Missing value*	41	-
**German citizenship**	**708**	
Yes	453	64%
No	255	36%
*Missing value*	*165*	-
**Duration in Germany (in years, if born abroad)**	**545**	MED = 21 years (IQR 9–30) MW = 21.51 years (SD = 13.12, range = < 1–71)
*Missing value*	*1*	-
**11 most common countries of origin (if born abroad)**	**537**	
Russia	53	9.9%
Syria	51	9.5%
Ukraine	51	9.5%
Iran	32	6%
Romania	28	5.2%
Turkey	27	5%
Kazakhstan	23	4.3%
Iraq	22	4.1%
Afghanistan	21	3.9%
Bulgaria	15	2.8%
France	15	2.8%
*Missing value*	*8*	-
**Educational level (ISCED)**	**703**	
High	486	69.1%
Medium	183	26%
Low	34	4.8%
*Missing value*	*170*	-
**Highest school-leaving qualification**	**690**	
General university entrance qualification	591	85.7%
General certificate of secondary education	88	12.8%
Primary school	5	0.7%
Still in school	3	0.4%
No school leaving qualification	3	0.4%
*Missing value*	*183*	-
**Highest educational attainment**	**674**	
Doctorate/PhD/Habilitation	30	4.6%
University - Master’s degree	249	36.9%
University - Bachelor’s degree	157	23.3%
University of Applied Sciences	31	4.6
Vocational college	66	9.8%
Completed apprenticeship	63	9.4%
Still in vocational training	34	5%
No professional qualification	44	6.5%
*Missing value*	*199*	-
**Is the highest educational attainment received abroad (if completed)?**	**596**	
Yes	256	43%
No	440	57%
*Missing value*	*0*	-
**Is the highest educational attainment recognized in Germany (if received abroad)?**	**256**	
Yes	160	62.5%
No	77	30.1%
On-going	19	7.4%
*Missing value*	*0*	-
Occupational status (additionally to interpreting)*	**708**	
Full-time	142	20.1%
Part-time	177	25%
Mini job (less than 15 h/week)	67	9.5%
Freelancer/self-employed	128	18.1%
Not employed, job-seeking	52	7.3%
Not employed, not job-seeking	33	4.7%
Retired	42	5.9%
No working permission	8	1.1%
Unable to work	7	1%
Full-time as interpreter and no other job	78	11%
*Missing value*	*165*	-

*Multiple answers were possible

#### Work-related variables

##### Interpreting experience, frequency and occupational status

At the time of the survey, 23.2% of the participants had been interpreting for less than 1 year. Those who had been interpreting for more than a year had on average *M =* 9.21 years of work experience (*SD =* 8.82) with a median of 6 years (*IQR =* 3–10). The longest-serving CIP has worked for a total of 55 years as an interpreter. Participants interpret on average *M* = 20.92 h (*SD =* 27.56) with a median of 10 h per month as CIPs and 75% of the sample do not exceed 30 h (*IQR =* 5–30 h). Most of them interpret every now and then (43.7%), 28.3% in part-time and 17% in full-time. A total of 11.8% are employed as CIPs, 50.8% are self-employed/freelance, and 2.7% are both. Just over one-third (34.4%) indicated that they interpret fully or additionally on a voluntary basis (unpaid).

##### Settings and type of interpreting

CIPs work on average in *M =* 4.17 different settings (*SD =* 2.06). Most of the time participants work on-site (*M = 82.76*%, *SD =* 24.75; *MED =* 90%), followed by telephone (*M* = 12.33%, *SD =* 19.82, *MED =* 5%) and video interpreting (*M =* 4.91%, *SD =* 13.39, *MED* < 0%).

##### Payment

While most participants (74.3%) reported getting often or always paid, 8.1% were paid sometimes and 17.7% were rarely or never paid. The average payment is *M =* 26.06 Euros (*SD =* 19.54) with a median of 20 Euros (*IQR =* 15–30 Euros). A total of 65.3% consider the payment too low, and 33.5% consider it appropriate. Among those who rate the payment to be inadequate, the desired payment is on average *M =* 40.67 Euros (*SD =* 23.27) with a median of 35 Euros per hour (*IQR =* 25–50).

##### Working languages

In total, 91.2% stated German as one of their working languages. Among those, 23.8% are German native speakers. Of those who are non-native speakers, 80.6% have a German language certificate according to the Common European Framework of Reference (GER), whereas 91.6% possess B2 or a higher level. The average number of working languages is *M* = 2.74 (*SD =* 1.01). In addition to German, the most common working languages are English (*n* = 227), Russian (*n* = 205) and Arabic (*n* = 161). All work-related variables are displayed in Table 2.

**Table 2:** Interpreting related work characteristics of the sample (*N* = 873)

	*n*	value
**Interpreting experience**	**852**	
Less than 1 year	198	23.2%
1 year or more	654	76.8% MED = 6 years (IQR 3–10) MW = 9.21 years (SD = 8.82, range = 1–55)
*Missing value*	*21*	-
**Working hours per month**	**839**	MED = 10 h (IQR 5–30) MW = 20.91 h (SD = 27.56, range = 1-170)
*Missing value*	*34*	-
**Occupational status as interpreter**	**854**	
Every now and then	373	43.7%
Part-time	242	28.3%
Full-time	145	17%
Other	94	11%
*Missing value*	*19*	-
**Employment status**	**854**	
Freelancer/self-employed	434	50.8%
Only or additionally on voluntary basis	294	34.4%
Employed	101	11.8%
Both	23	2.7%
Other	98	34.7%
*Missing value*	*19*	-
Settings*	**855**	
Authority	700	81.9%
Social service	634	74.2%
Education	610	71.4%
Health care	580	67.8%
Psychotherapy	356	41.6%
Legal service	283	33.1%
Police	222	20.7%
Court	177	26%
Other	98	11.5%
*Missing value*	*18*	-
**Number of interpreting settings**	**855**	MED = 4 settings (IQR 3–6) MW = 4.17 settings (SD = 2.06, range = 1–8)
1	88	10.3%
2	110	12.9%
3	165	19.3%
4	133	15.6%
5	131	15.3%
6	94	11%
7	59	6.9%
8	75	8.8%
*Missing value*	*18*	-
**Type of interpreting in %**	**829**	
On-site	829	MED = 90% (IQR 80–100) MW = 82.76% (SD = 24.75, range = 0-100)
Telephone	829	MED = 5% (IQR = 0–20) MW = 12.33% (SD = 19.82, range = 0-100)
Video	829	MED < 0% (IQR = 0–2) MW = 4.91% (SD = 13.39, range = 0-100)
*Missing value*	*44*	-
**Interpreting in German**	**761**	
Yes	694	91.2%
No	67	8.8%
*Missing value*	*112*	-
**German native language (if interpreting in German)**	**694**	
Yes	163	23.8%
No	521	76.2
*Missing value*	*10*	-
**Certificate for German language (non-native speaker)**	**521**	
No	101	19.4%
Yes	420	80.6%
A1	5	1.5%
A2	0	0%
B1	24	7%
B2	72	21.1%
C1	147	43%
C2	94	27.5%
*Missing value*	*78*	-
**Number of working language**	**761**	MED = 3 (IQR 2–3) MW = 2.74 (SD = 1.01, range = 1–6)
*Missing value*	*112*	-
10 most frequent working languages*	**761**	
English	227	-
Russian	205	-
Arabic	161	-
Persian	101	-
French	93	-
Ukrainian	74	-
Turkish	61	-
Spanish	51	-
Kurdish	45	-
Rumanian	38	-
*Missing value*	*112*	-
**Payment**	**780**	
Never	84	10.8%
Rarely	54	6.9%
Sometimes	63	8.1%
Often	137	17.6%
Always	442	56.7%
*Missing value*	*93*	-
**Amount of payment in EUR**		
Highest pay	690	MED = 25 Euros/h (IQR 15–48) MW = 36.53 Euros/h (SD = 33.28, range = 5-400)
*Missing value*	*183*	-
Lowest pay	687	MED = 15 Euros/h (IQR 12–25) MW = 19.75 Euros/h (SD = 13.94, range = 1-100)
*Missing value*	186	-
Average pay	690	MED = 20 Euros/h (IQR 15–30) MW = 26.06 Euros/h (SD = 19.54, range = 1-200)
*Missing value*	*183*	-
**Appropriateness of payment**	**777**	
Very low	208	26.8%
Somewhat low	299	38.5%
Adequate	260	33.5%
Somewhat high	8	1%
Very high	2	0.3%
*Missing value*	*96*	-
**Desired payment per hour (if not adequate)**	**515**	MED = 35 Euros/h (IQR 25–50) MW = 40.67 Euros/h (SD = 23.27, range = 0-150)
*Missing value*	*2*	-

*Multiple answers possible

##### Interpreting-related support services

The survey asked about specific support services available to CIPs (see Table 2.1 in the supplement files). A total of 43.5% reported having access to supervision. Among those who do not have or do not know if they have access to supervision, 43.9% would like to have it. Three-thirds of the sample (74.8%) have access to interpreting-specific training. Among those who stated that they do not have or do not know if they have access to any training, 69.8% desire to participate in such training. In response to the open question about further aspects of improving working conditions, the following were mentioned: better payment; more security regarding interpreting jobs; more information to prepare for interpreting jobs; professional organisation of CIPs; training of service providers; permanent employment as CIPs; and more appreciation and recognition of CIPs in general.

#### Qualification

##### Amount and content of training

In total, 69% of the participants reported that they had attended some kind of training on interpreting in the past (on-site or online). On average participants had *M =* 114.04 h of training (*SD =* 359.52) with a median of 25 h (*IQA =* 10–70). Among those who attended any kind of training, 29.4% participated in a training course with a final exam and successfully passed it. In most cases, the training was not specifically tailored to one setting, but rather generic (85.2%).

##### Attitude toward training

In total, 26.1% of respondents reported no need for interpreting-specific training, 34.6% expressed a partial need for training, and 39.3% indicated a need for training. The main reasons for not participating in (further) training are that training courses are not known, training is not considered worthwhile because no financial benefit is expected or because participants felt sufficiently experienced. Other reasons for not participating in (further) training provided in open questions are as follows: training offers do not match personal needs; lack of interest and motivation; excessive effort; training not suitable for certain languages; no certificate/no proof of participation; and lack of online training.

##### Self-reported interpreting competence

In terms of interpreting competence, the majority of participants perceive themselves as rather competent (46.8%) or very competent (28.1%). Of those who have never attended any training, 68.5% rated themselves as rather or very competent. All training-related data are displayed in Table 3.

**Table 3:** Training of the sample (*N* = 873)

	*n*	value
**Training received**	**725**	
Yes	500	69%
No	225	31%
*Missing value*	*148*	*-*
**Numbers of hours trained (if training received)**	**498**	MED = 25 h (IQR 10–70) MW = 114.04 (SD = 359.52, range = 1–3,000)
*Missing value*	*2*	*-*
Interpreting degree excluded	452	MED = 20 h (IQR 10–60) MW = 87.23 (SD = 293,24, range = 1–3,000)
*Missing value*	*1*	*-*
Interpreting degree	44	MED = 75 h (IQR 36.35–375.00) MW = 389.43 (SD = 707.72, range = 2–3,000)
*Missing value*	*3*	*-*
Without exam	352	MED = 20 h (IQR 8–40) MW = 38.07 (SD = 71.48, range = 1-800)
*Missing value*	1	*-*
With exam	144	MED = 80 h (IQR 30–200) MW = 299.74 (SD = 621.24, range = 2–3,000)
*Missing value*	*3*	*-*
**Exam passed (if training received)**	**500**	
Yes	147	29.4%
No	353	70.6%
*Missing value*	*0*	*-*
Type of exam* (if exam passed)	147	
Interpreting degree	47	32%
State-certified interpreter	22	15%
Chamber of Commerce	19	12.9%
Other	85	57.8%
*Missing value*	*0*	*-*
**Sworn-In for interpreting at court**	**725**	
Yes	93	12.8%
No	632	87.2%
*Missing value*	*148*	*-*
Topics covered in training* (if training received)	**500**	
Roles of interpreters	357	71.4%
Ethical principles in interpreting	270	54%
Interpreting techniques	254	50.8%
Self-care & mental health	207	41.4%
Legal aspects of interpreting	177	35.4%
Telephone/video interpreting	139	27.8%
Note taking	130	26%
Entrepreneurial skills	86	17.2%
Setting specific training		
Generic training	426	85.2%
Authority	193	38.6%
Psychotherapy	173	34.6%
Education	170	34%
Social service	169	33.8%
Health care	164	32.8%
Legal	97	19.4%
Court	65	13%
Police	60	12%
Other topics	11	2.2%
*Missing value*	*0*	*-*
**Subjective need to receive trained**	**725**	MED = 3 (IQR 2–4) MW = 3.22 (SD = 1.23)
Not at all needed	79	10.9%
Rather not needed	110	15.2%
Partly needed	251	34.6%
Rather needed	146	20.1%
Highly needed	139	19.2%
*Missing value*	*148*	*-*
Barriers to attend training*		
Trainings not known	725	MED = 3 (IQR 1–4) MW = 2.74 (SD = 1.51)
Not worth it, no financial benefit	725	MED = 3 (IQR 1–4) MW = 2.31 (SD = 1.26)
No need, as sufficient experience already	725	MED = 3 (IQR 2–3) MW = 2.47 (SD = 1.36)
Distance	725	MED = 2 (IQR 1–3) MW = 2.75 (SD = 1.38)
Family or work commitments	725	MED = 2 (IQR 1–3) MW = 2.64 (SD = 1.21)
Not worth it, interpreting too rarely	725	MED = 2 (IQR 1–3) MW = 2.21 (SD = 1.22)
Have already participated in a sufficient number of trainings	725	MED = 2 (IQR 1–3) MW = 1.94 (SD = 1.15)
*Missing value*	*148*	*-*
**Interpreting competence**	**725**	MED = 3 (IQR 2–4) MW = 3.01 (SD = 0.77)
Not competent	2	0.3%
Rather not competent	10	1.4%
Averagely competent	170	23.5%
Rather competent	339	46.8%
Very competent	204	28.1%
*Missing value*	*148*	*-*

*Multiple answers were possible

#### Mental health status

##### General psychosocial distress, anxiety and depression

In total, 51.1% reported a score above the cut-off of 4 on the NCCN distress scale, showing a moderate to severe level of general psychosocial distress. The average score is *M =* 3.78 (*SD =* 2.78). Concerning depressive and general anxiety symptomatology, measured with the PHQ-2 and GAD-2, the average scores are *M* = 0.89 (*SD =* 1.21) and *M =* 0.89 (*SD =* 1.16), respectively. In total, 9.7% reported a score above the cut-off of 3 indicating high depressive symptoms and 7.7% reported a score above the cut-off of 3 indicating high anxiety symptoms.

##### Psychosocial distress related to interpreting

Concerning the psychosocial distress related to interpreting, 36.1% of the sample reported a score above the cutoff of 4 on the NCCN distress scale, showing a moderate to severe level of general psychosocial distress. The average score is *M =* 2.85 (*SD =* 2.6). Distressing factors that were additionally mentioned in open answers are displayed in Table 4.

**Table 4:** Distressing factors

Structural/institutional factors	Interpersonal factors	Interpreting situation
• Poor compensation for CI services. • Precarious financial situation of CIPs. • Lack of planning certainty with regard to interpreting jobs and demanded high flexibility. • Lack of training of service providers to work with CIPs. • Lack of training of CIPs, leading to low confidence in own interpreting skills.	• Lack of respect, appreciation, and recognition on the part of the service provider and user. • Perceived discrimination, such as racism and sexism, on the part of the service provider and user.	• Emotional distress due to translating the details of service users’ stressful/traumatic experiences. • High time pressure. • Overlapping talks. • Cultural conflicts. • Role conflicts and difficulties in maintaining professional boundaries.

*Note* Factors were reported in open answers by participants

### Differences depending on the training of community interpreters

To explore differences depending on the training of CIPs, we conducted dependent t-tests between participants who have participated in any kind of training and people who have not. In this sample, people who have participated in any training are significantly older (t (382) = 2.57, p-value = 0.011), stay for a longer period in Germany (t (382) = 2.38, p-value = 0.018), perceive themselves as more competent (t (382) = 2.01, p-value = 0.045), are more experienced (t (382) = 2.53, p-value = 0.012), work in more settings (t (382) = 4.64, p-value < 0.001), are less distressed in general (t (382) = -2.44, p-value = 0.015), and less distressed regarding interpreting (t (382) = -2.2, p-value = 0.029). Differences in terms of gender, education, interpreting frequency, frequency and amount of payment, and need to receive training were not found.

### Relevant factors for participating in interpreting-specific training

To explore how these variables contribute to the likelihood of partaking in interpreting-specific training, we conducted a logistic regression analysis. We included the identified factors that differed between participants with and without training in the regression model. Beforehand, we used the Pearson statistic to test for multicollinearity, whereby none of the predictors correlated more strongly than *r* = .603. Most variables are not associated with participation in interpreting specific training in this regression model. Only the number of settings was found to be significantly associated with participating in interpreting-specific training when the remaining six variables were controlled for (OR: 1.31, CI 95%: 1.15–1.48). The full model explains 11.7% of the variance in whether CIPs in this study have participated in interpreting-specific training (Table 5).

**Table 5:** Logistic regression analyses for participation in interpreting specific training (*N* = 873)

	Model 1 (*n* = 420)		
Independent variable	OR	95% CI	*p*-value
1) Age	1.012	0.987-1.038	0.337
2) Duration of stay in Germany	1.007	0.984-1.031	0.545
3) Interpreting competence	1.212	0.892-1.647	0.218
4) Interpreting experience in years	0.987	0.952-1.024	0.496
5) Number of settings	**1.305**	1.154–1.475	< 0.001
6) NCCN Distress general	0.958	0.870-1.055	0.386
7) NCCN Distress re interpreting	0.943	0.852-1.043	0.252
Nagelkerkes R^2^	0.117		

### Correlates of psychosocial distress regarding interpreting

In a first step, Spearman’s correlation analyses were calculated to identify possible relationships between psychological distress regarding interpreting and sociodemographic, work-related training and mental health variables (see Table 6). When examining the correlates of psychological distress regarding interpreting, a significant association can be observed with the frequency of interpreting (*r*_*s*_ = 0.243, p-value < 0.001), meaning that individuals interpreting more frequently experience higher psychosocial distress. Additionally, significantly negative relationships were found with length of stay (*r*_*s*_ = − 0.105, p-value < 0.05), meaning that participants who stay for a longer period of time in Germany show less psychosocial distress regarding interpreting. Frequency and satisfaction with payment (*r*_*s*_ = − 0.155, p-value < 0.001 and − 0.124, p-value < 0.01) were also significantly negatively associated, meaning that CIPs who are paid more often and are more satisfied with their pay show less psychological distress regarding interpreting. The strongest positive relationships were found with depressive and anxiety symptoms as well as psychological distress in general (*r*_*s*_ = 0.306, 0.259, 0.516, p-value < 0.001, respectively), suggesting that individuals with more severe depressive and anxiety symptoms and higher psychological distress in general experience more distress regarding interpreting. No significant correlations could be found with interpreting experience or the need to receive training.

**Table 6:** Correlations of sociodemographic and work-related variables, training and mental health outcomes (*N* = 873)

	1	2	3	4	5	6	7	8	9	10	11	12	13	14	15	16	
1) Age	1.000																
2) Education	0.154***	1.000															
3) Length of stay	0.601***	− 0.085*	1.000														
4) Training hours	0.158***	0.054	0.131**	1.000													
5) Interpreting competence	0.089*	0.112**	0.165***	0.250***	1.000												
6) Interpreting frequency	< 0.000	0.030	0.021	0.089*	0.270***	1.000											
7) Experience	0.415***	0.076	0.362***	0.237***	0.281***	0.249***	1.000										
8) Number of settings	0.158***	0.051	0.178***	0.344***	0.257***	0.281***	0.308***	1.000									
9) Frequency of payment	0.043	0.057	0.143**	0.201***	0.149***	0.021	− 0.089*	0.116**	1.000								
10) Amount of payment	0.129**	0.202***	0.157**	0.325***	0.300***	0.238***	0.346***	0.264***	0.279***	1.000							
11) Appropriate-ness of payment	− 0.085*	0.021	− 0.025	− 0.049	− 0.153***	− 0.092*	− 0.089*	− 0.182***	0.156***	0.081*	1.000						
12) Need to receive training	− 0.083*	− 0.031	− 0.079	0.004	− 0.124**	− 0.057	− 0.118**	− 0.089*	− 0.057	− 0.088*	− 0.024	1.000					
13) PHQ-2 Depression	− 0.091*	− 0.024	− 0.116**	0.023	− 0.140***	0.003	− 0.041	− 0.042	− 0.077*	− 0.046	− 0.090*	0.066	1.000				
14) GAD-2 Anxiety	− 0.155***	− 0.027	− 0.177***	− 0.001	− 0.114**	− 0.035	− 0.101*	− 0.043	− 0.048	0.009	− 0.020	0.122**	0.518***	1.000			
15) NCCN Distress general	− 0.131***	− 0.014	− 0.035	− 0.090*	− 0.024	0.019	− 0.089*	− 0.059	− 0.058	− 0.011	− 0.084*	0.044	0.390***	0.464***	1.000		
16) NCCN Distress re interpreting	− 0.073	− 0.007	− 0.105*	− 0.043	− 0.041	0.243***	0.020	− 0.025	− 0.155***	0.042	− 0.124**	0.021	0.306***	0.259***	0.516***	1.000	

*Note* *p < .05; **p < .01; ***p < .001

### Relevant factors for interpreting specific distress

In a second step, we conducted exploratory multiple hierarchical stepwise regression analyses to examine the variables that significantly correlated with psychosocial distress regarding interpreting in more detail. No evidence of multicollinearity was found regarding the variance inflation factor. The residual plots showed homoscedasticity and normal distributions of the residuals. There were four hierarchical regression models whose variables are displayed in Table 7. The first model only included general distress as an independent variable and explained 28.4% of the variance. The second model added 1.7% and the third another 1.9% explained variance. The final model, which included perceived distress in general (β = 0.433, p-value < 0.001), interpreting frequency (β = 0.013, p-value < 0.001), depressive symptoms (β = 0.293, p-value < 0.001) and appropriateness of payment (β = − 0.324, p-value = 0.004), added 1.0% explained variance, leading to R^2^ = 0.326. This means that being more distressed in general, interpreting more often, having more severe depressive symptoms and being less satisfied with the payment emerged as significant correlates with perceived distress regarding interpreting and accounted for 32.6% of the variance in the present study.

**Table 7:** Hierarchical regression for interpreting specific distress

Variable	B	SE B	β	*p*
**Step 1** ^**a**^				
Constant	0.998	0.157		< 0.001
NCCN distress general	0.496	0.034	0.533***	< 0.001
**Step 2** ^**b**^				
Constant	0.767	0.168		< 0.001
NCCN distress general	0.486	0.034	0.523***	< 0.001
Interpreting frequency	0.012	0.003	0.132***	< 0.001
**Step 3** ^**c**^				
Constant	0.650	0.168		< 0.001
NCCN distress general	0.437	0.035	0.470***	< 0.001
Interpreting frequency	0.013	0.003	0.141***	< 0.001
PHQ2 Depression	0.311	0.079	0.149***	< 0.001
**Step 4** ^**d**^				
Constant	1.360	0.298		< 0.001
NCCN distress general	0.433	0.035	0.465***	< 0.001
Interpreting frequency	0.013	0.003	0.139***	< 0.001
PHQ2 Depression	0.293	0.079	0.140***	< 0.001
Appropriateness payment	− 0.324	0.113	− 0.102**	0.004

*Note* *p < .05; **p < .01; ***p < .001; ^a^R² = 0.284, *p* < .001 ^b^ΔR² = 0.017, *p* < .001 ^c^ΔR² = 0.019, *p* < .001 ^d^ΔR² = 0.010, *p* = .004

### Correlates of self-reported interpreting competence

Spearman’s correlation analyses were calculated to identify possible relationships between interpreting competence and sociodemographic, work-related training and mental health variables (see Table 6). Significant positive correlations were found with age (*r*_*s*_ = 0.089, *p* < .05), education (*r*_*s*_ = 0.112, *p* < .01) and length of stay in Germany (*r*_*s*_ = 0.165, *p* < .001), meaning that CIPs who are older, have a higher educational level and stay for a longer period of time in Germany perceive themselves as more competent. Significant positive associations were also found with hours of training (*r*_*s*_ = 0.250, *p* < .001), frequency of interpreting (*r*_*s*_ = 0.270, *p* < .001), overall interpreting experience (*r*_*s*_ = 0.281, *p* < .001), number of different interpreting settings (*r*_*s*_ = 0.257, *p* < .001), frequency of payment (*r*_*s*_ = 0.149, *p* < .001) and amount of payment received for interpreting services (*r*_*s*_ = 0.300, *p* < .001). This suggests that participants who have completed more hours of training, interpret more frequently, are more experienced, work in many different settings, and receive more often as well as a higher amount of payment perceive themselves as more competent regarding interpreting. Furthermore, self-reported interpreting competence exhibited significant negative correlations with the appropriateness of payment (*r*_*s*_ = − 0.153, *p* < .001), the subjective need for training (*r*_*s*_ = − 0.124, *p* < .001), depressive symptoms (*r*_*s*_ = − 0.140, *p* < .001) and anxiety symptoms (*r*_*s*_ = − 0.114, *p* < .01), meaning that individuals who perceive themselves as more competent are less satisfied with their payment, have a lower need for training, and show fewer depressive and anxiety symptoms.

### Relevant factors for interpreting competence

Following the correlational analysis, exploratory multiple hierarchical stepwise regression analyses were conducted to examine the variables that significantly correlated with interpreting competence in more detail. No evidence of multicollinearity was found regarding the variance inflation factor. The residual plots showed homoscedasticity and normal distributions of the residuals. There were five hierarchical regression models whose variables are displayed in Table 8. The first model only included the frequency of interpreting as an independent variable and explained 6.4% of the variance. The second model added 4.5%, the third another 3.3%, and the fourth 1.8% explained variance. The final model, including interpreting frequency (β = 0.006, p-value < 0.001), anxiety symptoms (β = − 0.125, p-value < 0.001), amount (β = 0.007, p-value < 0.001), frequency (β = 0.131, p-value = 0.001) and appropriateness of payment (β =- 0.127, p-value = 0.005), added 1.7% explained variance, leading to R^2^ = 0.168. This means that interpreting more often, having less severe anxiety symptoms, receiving a higher payment, getting paid more often and perceiving the payment as less appropriate emerged as significant correlates with perceived competence regarding interpreting and accounted for 16.8% of the variance in this study.

**Table 8:** Hierarchical regression for interpreting competence

Variable	B	SE B	β	*p*
**Step 1** ^**a**^				
Constant	1.948	0.046		< 0.001
Frequency of interpeting	0.006	0.001	0.258***	< 0.001
**Step 2** ^**b**^				
Constant	2.074	0.053		< 0.001
Frequency of interpeting	0.006	0.001	0.247***	< 0.001
GAD-2 anxiety	− 0.133	0.030	− 0.212 ***	< 0.001
**Step 3** ^**c**^				
Constant	2.864	0.076		< 0.001
Frequency of interpeting	0.006	0.001	0.232***	< 0.001
GAD-2 anxiety	− 0.127	0.030	− 0.202 ***	< 0.001
Amount of payment	0.008	0.002	0.183***	< 0.001
**Step 4** ^**d**^				
Constant	2.386	0.185		< 0.001
Frequency of interpeting	0.006	0.001	0.232***	< 0.001
GAD-2 anxiety	− 0.120	0.030	− 0.191 ***	0.002
Amount of payment	0.007	0.002	0.152***	0.005
Frequency of payment	0.115	0.041	0.137**	< 0.001
**Step 5** ^e^				
Constant	2.567	0.195		< 0.001
Frequency of interpeting	0.006	0.001	0.232	< 0.001
GAD-2 anxiety	− 0.125	0.030	− 0.199	< 0.001
Amount of payment	0.007	0.002	0.166	< 0.001
Frequency of payment	0.131	0.041	0.157	0.001
Appropriateness of payment	− 0.127	0.046	− 0.134	0.005

*Note* *p < .05; **p < .01; ***p < .001; ^a^R² = 0.064, *p* < .001 ^b^ΔR² = 0.045, *p* < .001 ^c^ΔR² = 0.033, *p* < .001 ^d^ΔR² = 0.018, *p* = .005 ^e^ΔR² = 0.017, *p* = .005

### Differences between interpreting settings

Since participants could state multiple interpreting settings in the present study, variables of interest were dichotomized, afterwards, crosstabs and multiple logistic regressions were performed to identify differences between the eight settings of health care, psychotherapy, social service, education, authority, legal service, police and court. The settings were examined regarding the selected variables: age, gender, education, interpreting experience, frequency of interpreting, qualification and interpreting competence and frequency and amount of payment. Individuals working in court emerged as a subgroup, significantly associated with being older (> median 43 years) (OR: 2.16, CI 95%: 1.47–3.19, p-value < 0.00); having more work experience (> median 6 years) (OR: 3.73, CI 95%: 2.24–6.21, p-value < 0.00); being trained (OR: 1.83, CI 95%: 1.1–3.07, p-value = 0.02) and receiving a payment above 20 Euros (median) (OR: 6.09, CI 95%: 3.68–10.06, p-value < 0.00). The results are displayed in more detail in the supplement files.

## Discussion

### Selected personal characteristics of community interpreters: gender, migration history, education

Although the total population of CIs in Germany remains unknown, the study findings provide preliminary evidence that the interpreting profession might be predominantly female. Almost three-quarters of the participants in this study identified as women. Similar gender distributions were also found in other studies [42, 47, 67]. CI is largely characterized by its caring and social nature, placing it in line with other caring professions, such as social work, nursing and psychology, which are dominated by females [67, 68].

Overall, more than 77% of the sample has a first-generation migration history and another 12% has a second-generation migration history. The main reasons for immigrating to Germany were family and political, legal or humanitarian reasons, accounting for approximately 39% and 28% of the participants who were born abroad respectively. In addition, other studies revealed that 25–27% of CIPs have a personal history of flight [46, 47]. The foreign-born participants in the current study have lived in Germany for an average of 21.5 years at the time of the survey and more than one-third of the total sample does not have German citizenship. In comparison, among all migrants in Germany, 53% do not hold German citizenship [69].

In line with previous studies [46, 47], the level of education in this sample was high according to the ISCED [66]. Almost 65% of the CIPs in this study had at least a Bachelor’s university degree (5.4% completed an interpreting degree). However, it should be emphasized that 43% of the participants who have completed an educational qualification received their highest educational attainment abroad and almost one-third of them stated that their degree is not recognized in Germany. Based on the results of the present study it can be assumed that a significant proportion of those working as CIPs may in fact have acquired other professional qualifications. As shown in previous studies, if migration is not primarily due to education or work reasons, the (non-)recognition of foreign-acquired qualifications is one of the biggest obstacles for first-generation migrants to access the labor market of the country of arrival [70]. Two-thirds of the participants in this study reported full-time or part-time employment or self-employment/freelance work in addition to working as a CIP, indicating that CI functions most often as a supplementary occupation. The reasons for this should be investigated in more detail in future studies.

### Mental health status of community interpreters

More than 50% of this study’s sample reported moderate or severe psychosocial distress in general, which is in line with other findings [46, 47, 49, 71]. For instance, Geiling et al. (2022) showed increased psychological distress among CIPs in refugee care settings in Germany [46]. Jurado et al. (2017) reviewed factors associated with psychological distress in migrant populations around the world. Female gender and forced and poorly planned migration, such as migration for political, legal, or humanitarian reasons, are associated with a higher level of distress [72]. Mylord et al. (2023) specifically demonstrated that migrants without German citizenship have less social support, less psychological resilience, and experience more discrimination, which could affect their mental health [73]. Such risk factors are also predominantly present in this study’s sample. In contrast, almost 45% of the participants in the current study are employed full- or part-time. The CIPs who were born abroad have been residing in Germany on average for 21.5 years and the vast majority possess medium levels of German language skills. These factors have been found to be associated with lower levels of psychosocial distress in migrant populations [72].

While most CIPs in this study reported no or only low levels of depression and anxiety symptoms, previous research found significant differences in anxiety and depression among CIPs [46] and a significantly higher prevalence of secondary traumatization or posttraumatic stress disorder in comparison to the general German population [46, 47, 71]. However, most of these studies focus on interpreting in mental health or refugee settings, where psychological strain can be particularly high. Future studies could build on these findings by employing different instruments, e.g. General Health Questionnaire [74], to assess various aspects of mental health.

### Interpreting related psychological distress and its associated factors

Concerning interpreting-related psychological distress and its associated factors, this study’s findings complement previous research [42, 46]. The present study found that more than one-third of the participants perceive interpreting in community settings as psychologically distressing, emphasizing the need for further psychosocial support services for CIPs. The frequency of interpreting was positively associated with psychological distress regarding interpreting, meaning that participants who reported higher levels of psychosocial distress interpreted more frequently. Furthermore, the correlation analysis showed that interpreters who get paid more often and are more satisfied with the payment are less distressed. Depression, anxiety and general distress were also positively linked to distress regarding interpreting.

In the regression analyses, having more stress in general and more severe depressive symptoms, interpreting more often and being less satisfied with the payment for interpreting were associated with higher perceived distress regarding interpreting. This suggests that CIPs’ overall psychological well-being as well as their workload and working conditions, such as the payment, play a significant role in CIPs’ experience of distress related to interpreting. This is in line with previous studies, reporting dissatisfaction with pay to be linked with higher work-related exhaustion [46]. Moreover, higher workload was identified as a risk factor in a recent systematic review of the mental health and work experiences of interpreters in mental health care settings [42]. Although it must be taken into account that no causal conclusions can be drawn based on our study findings, the open responses in our study indicate that precarious working conditions, such as poor compensation, lack of planning certainty and high flexibility, could negatively impact CIPs’ psychosocial distress.

Furthermore, in line with previous literature [25, 29, 45–49], participants described in open questions interpersonal aspects as distressing, such as lack of respect, appreciation, and recognition as well as discrimination. In the interpreting situation, factors such as role conflicts and difficulties in maintaining professional boundaries were reported as distressing. In-depth research on psychosocial distress and its determinants among CIPs and across settings, e.g. by measuring further job-related data, such as professional recognition, relationships with other professional groups and experienced discrimination; using qualitative methods or a longitudinal study design is needed for future studies.

### Community interpreters’ working conditions

The results indicate that the vast majority of participants in this study primarily work as freelance/self-employed CIPs, with a smaller percentage (15%) reporting employment in this capacity. One-third (34%), interpret solely or additional on a voluntary basis (unpaid), which underlines the caring nature of CI. In a study by Kindermann et al. (2017), the number of volunteer CIPs was almost twice as high, at 67% [47]. CIPs in the present study interpret a median of 10 h per month, and 75% of the sample does not exceed 30 h per month. A survey conducted in 2019 by a professional association for interpreters in Germany showed similar results. Among those who interpret part-time, 75% interpret 2–3 times a month or less [51].

Since there is no legal entitlement to interpretation in most community settings in Germany, there is also no legally regulated remuneration for CIPs leading to a great variation of payment [39]. Low and dissatisfying payment can be associated with work-related exhaustion [46] and undermine CIPs’ professionalism [75, 76]. While 11% of the participants in the present study indicate that they get never paid, most of them reported getting sometimes, often or always paid with a median rate of 20 Euro per hour. Although the majority of participants were not satisfied with the payment, as 65% considered it to be too low, the desired payment was still relatively low, with a median of 35 Euros. Sample calculations for CIPs in Germany show that even an hourly rate of 60 Euros only generates an income that is close to the poverty threshold [76]. It would be interesting to investigate CIPs’ attitude toward payment in more detail in future studies and explore potential influencing factors, such as the perceived status and recognition of the CI profession or caring and voluntary attitudes of CIPs.

Previous studies described that low and insecure working conditions could affect CIPs’ commitment to interpreting, resulting in interpreting often being only fitted in between other (work) commitments [77, 78]. This is supported by the results of this study. Based on the study results, it can be assumed that CI in Germany currently may be more commonly pursued on the side or on a voluntary basis rather than as a full-time profession. This aligns with previous findings that only a small percentage of interpreters working in Germany derive their main income from interpreting in community settings, such as authorities, health and social services (3%) or the judicial sector (6%) [51].

As often discussed in the literature [21, 32, 39, 79], individuals who interpret at court emerged as a subgroup in the present study for a number of sociodemographic and work-related variables. Court interpreting is one of the few settings in Germany in which standards are already established, for instance, remuneration is regulated by law. Based on our findings, this subgroup tends to be older, more experienced, have a higher probability of being trained, have received more training and completed a final exam more often, and are more likely to get always and also higher paid. Based on our study results, setting-related differences should be further investigated.

### Qualification, interpreting competence and its associated factors

Qualification is widely recognized not only as a crucial component in ensuring the delivery of high-quality interpreting services but also as a fundamental factor in shaping a profession and improving its recognition and status [21, 80, 81]. While a significant proportion of the participants in this study reported previous participation in some form of training (69%), the median hours of received training are relatively low at 25 h. Among those who had undergone training, 75% of the sample had received no more than 70 h of training.

In this study, we found that CIPs who have attended any kind of interpreting-specific training tend to be older, stay for a longer time in Germany, perceive themselves as more competent, are more experienced, work in more settings and are less distressed in general and also perceive interpreting as less distressing. However, the regression analyses showed that only the number of interpreting settings was associated with CIPs partaking in training. Thus, CIPs who worked in a greater variety of settings were more likely to have undergone interpreting-specific training. However, as no causal conclusions can be drawn based on the present study, it is not possible to say whether participation in training leads to CIPs working in more settings or vice versa. This, and other influencing factors that may not have been taken into account in this study, should be investigated in future studies.

Although the majority expressed a subjective need for training, there were notable barriers to attending such training. These barriers include the perception that participation in (further) training is not worthwhile due to a lack of financial benefits. Similarly, Ozolins (2004) reported that CIPs’ interest in training is strongly related to their working conditions and that interpreters might only partake in such training if it leads to higher remuneration [77, 82]. Another barrier identified in this study was a lack of training opportunities. This could be attributed to the absence of accessible local training and certification programs, as well as a widely varied and potentially confusing training landscape. Conversely, it may also stem from a lack of knowledge about existing training offerings in the CI community. For future studies, the use of qualitative methods could be useful to investigate barriers to attending training and CIPs’ attitudes toward training. The implementation of national and legally anchored minimum requirements for the qualification of CIPs could be beneficial in terms of increasing the level of qualification in Germany in the long term [40]. Since participants in this study work on average in four different settings, it is recommended to establish generic training and certification programs rather than tailoring training to specific individual settings or client groups.

The majority of participants in this study assessed their subjective interpreting competence as rather high. The regression analyses demonstrated that CIPs’ interpreting more frequently, having less severe anxiety symptoms, receiving higher pay, getting paid more often and perceiving the payment as less appropriate perceived themselves as more competent. Surprisingly, training was not associated with perceived competence in the regression analysis. In line, among those who never attended any kind of training, 69% perceived themselves as rather or very competent and the belief that no (further) training is needed because one already has sufficient interpreting experience emerged as one of the main barriers to attending training in this study. However, when looking at the differences between participants being somehow trained vs. participants not being trained, it could be found that the participants who have participated in training also reported higher interpreting competence.

A study conducted by Fitzmaurice (2020) among educational interpreters working in public schools showed that the least skilled interpreters tend to overestimate their interpreting skills and that more skilled interpreters underestimate their interpreting competence. One possible explanation could be the effect that individuals with limited knowledge lack the expertise to accurately evaluate their own performance or level of competence. Such individuals may mistakenly believe they possess a high level of expertise due to their lack of awareness about the complexities and nuances of a task or topic, also called the Kruger and Dunning effect [83]. It also emphasizes the need for CIPs to get trained to bridge the gap between perceived and actual competence. Through training, CIPs may gain a more comprehensive understanding of the demands of interpreting, which can lead to a more accurate self-assessment of their competencies. A more in-depth understanding of factors influencing CIPs’ subjective competence may be achieved through the use of qualitative methods in future studies.

### Strengths and limitations of the study

Despite the large sample size, whether the sample represents the CIP population in Germany is unclear. Since working as a CIP is not an officially recognized occupation in Germany, no national statistics exist and it is unknown how many people are actually working as CIPs in Germany. Therefore, it is not known if the results of this survey are generalizable. Moreover, the generalizability of the study’s results is potentially compromised due to systematic biases that may arise from the use of convenience snowball sampling and online survey methods. By choosing an online survey, it could not be ruled out that some people not belonging to the target population might have participated. While dichotomization facilitated data analysis and proved useful for investigating differences between interpreting settings, it may have resulted in a loss of detail for some variables. This limitation implies that certain nuances or variations within the variables might have been overlooked, potentially limiting the depth of understanding. When interpreting the results, it must be taken into account that no causal statements can be made based on the data collected. To assess the direction of the associations found in this study, longitudinal studies are recommended for future studies.

The main strength of this study is its large sample size, indicating that a substantial number of the CIP population was included in the research. Furthermore, the national study explored a wide range of variables across interpreting settings and client groups, resulting in a comprehensive overview of CI in Germany. No other study could be found either nationally or internationally that is comparable in terms of its scope and sample size in the field of CI. This broad examination of aspects also allowed for a more thorough exploration of potential factors associated with participation in interpreting training, interpreting competence and perceived distress regarding interpreting. The results can guide future research by narrowing the focus to the variables identified as relevant in this study. Moreover, including individuals from the target group, as well as other significant stakeholders, in the development of the study design, questionnaire, and recruitment processes is a notable strength of this study. This approach enhances the relevance and validity of the study by incorporating the perspectives and insights of those directly affected by the research topic.

### Conclusion and implications

Despite increasing recognition in Germany of CI as a profession requiring legal and ethical regulation, along with a formal qualification including training and certification, this study uncovers a notable absence of formal training and certification among most CIPs. Moreover, CI activities were found to be predominantly conducted on-site and often carried out voluntarily (unpaid). Only a small proportion of the participants are employed and work as CIPs full-time. It can be assumed that precarious working conditions, particularly low compensation, may play a role in preventing many individuals from pursuing full-time employment in CI. Furthermore, this study’s findings indicate that CIPs can be considered a vulnerable group, which becomes evident, for instance, by the reported high level of general distress, reasons for migration, the post-migration stressors, such as lack of German citizenship, and precarious working conditions.

As highlighted previously by Pöllabauer (2012) and others, CI often receives scant recognition in the public eye and is not regarded as a professional service, which may reflect broader societal attitudes toward equal access, civil rights, and immigration [25, 84, 85]. This lack of acknowledgement, coupled with the absence of legal regulations regarding qualifications and funding for CI services, as well as the prevalence of poor working conditions and low wages, could be considered as repercussions of this undervaluation [32]. To further promote the professionalization of CI in Germany, it is imperative to establish national, legally binding qualification standards for CIPs, as well as implement uniform and comprehensive training programs and certification procedures. Moreover, overarching and formalized funding and organization of CI services must be established.

### Electronic supplementary material

Below is the link to the electronic supplementary material.

Supplementary Material 1

Supplementary Material 2

## Data Availability

Data included in this report are available on request. Please contact Saskia Hanft-Robert: s.hanft-robert@uke.de.

## References

[1] Poushter J, Fetterolf J, Tamir C. A changing world: global views on diversity, gender equality, family life and the importance of religion. Pew Research Center. 2019.

[2] McAuliffe M, Oucho LA (2024). World Migration Report 2024.

[3] Mösko MO, Gil-Martinez F, Schulz H (2023). Cross-cultural opening in German outpatient Mental Healthcare Service: an exploratory study of structural and procedural aspects. Clin Psychol Psychother.

[4] Groene OR, Huelmann T, Hampe W, Emami P. German physicians and medical students do not represent the population they serve. Healthcare. 2023;11(1662). 10.3390/healthcare11121662 PMC10298415 37372780

[5] Al Shamsi H, Almutairi AG, Al Mashrafi S, Al Kalbani T (2020). Implications of Language Barriers for Healthcare: a systematic review. Oman Med J.

[6] Hunter-Adams J, Rother HA (2017). A qualitative study of language barriers between South African health care providers and cross-border migrants. BMC Health Serv Res.

[7] Ohtani A, Suzuki T, Takeuchi H, Uchida H (2015). Language barriers and Access to Psychiatric Care: a systematic review. PS.

[8] Olani AB, Olani AB, Muleta TB, Rikitu DH, Disassa KG. Impacts of language barriers on healthcare access and quality among Afaan Oromoo-speaking patients in Addis Ababa, Ethiopia. BMC Health Serv Res. 2023;23(39). 10.1186/s12913-023-09036-z PMC9843916 36647040

[9] Satinsky E, Fuhr DC, Woodward A, Sondorp E, Roberts B (2019). Mental health care utilisation and access among refugees and asylum seekers in Europe: a systematic review. Health Policy.

[10] Suphanchaimat R, Kantamaturapoj K, Putthasri W, Prakongsai P. Challenges in the provision of healthcare services for migrants: a systematic review through providers’ lens. BMC Health Serv Res. 2015;15(390). 10.1186/s12913-015-1065-z PMC4574510 26380969

[11] Miteva D, Georgiadis F, McBroom L, Noboa V, Quednow BB, Seifritz E et al. Impact of language proficiency on mental health service use, treatment and outcomes: ‘Lost in translation’. Compr Psychiatr. 2022;114(152299). 10.1016/j.comppsych.2022.152299 35220037

[12] Borde T (2018). Kommunikation Und Sprache: Herausforderungen Und Chancen Einer diversitätsgerechten Gesundheitsversorgung. Gynäkologische Endokrinologie.

[13] Bauer AM, Alegría M (2010). Impact of Patient Language Proficiency and Interpreter Service Use on the quality of Psychiatric Care. Syst Rev.

[14] Jaeger FN, Pellaud N, Laville B, Klauser P. Barriers to and solutions for addressing insufficient professional interpreter use in primary healthcare. BMC Health Serv Res. 2019;19(753). 10.1186/s12913-019-4628-6 PMC6815061 31653211

[15] Karliner LS, Jacobs EA, Chen AH, Mutha S (2007). Do Professional interpreters improve clinical care for patients with Limited English proficiency? A systematic review of the literature. Health Serv Res.

[16] Smith J, Swartz L, Kilian S, Chiliza B (2013). Mediating words, mediating worlds: interpreting as hidden care work in a South African psychiatric institution. Transcult Psychiatry.

[17] Heath M, Hvass AMF, Wejse CM (2023). Interpreter services and effect on healthcare - a systematic review of the impact of different types of interpreters on patient outcome. J Migration Health.

[18] ten Thije JD, Singleton D, Aronin L (2018). Receptive multilingualism. Twelve lectures on multilingualism.

[19] Thonon F, Perrot S, Yergolkar AV, Rousset-Torrente O, Griffith JW, Chassany O (2021). Electronic Tools to Bridge the Language Gap in Health Care for people who have migrated. Syst Rev.

[20] Cox A, Rosenberg E, Thommeret-Carrière AS, Huyghens L, Humblé P, Leanza Y (2019). Using patient companions as interpreters in the Emergency Department: an interdisciplinary quantitative and qualitative assessment. Patient Educ Couns.

[21] Roberts RP, Carr SE (1997). Community Interpreting today and tomorrow. The critical link: interpreters in the community.

[22] Pöchhacker F (1999). Getting Organized’: the evolution of Community Interpreting. INTP.

[23] Gentile A, Ozolins U, Vasilakakos M (1996). Liaison Interpreting: a handbook.

[24] Pokorn NK, Mikolič Južnič T (2020). Community interpreters versus intercultural mediators: is it really all about ethics?. TIS.

[25] Pöllabauer S, Chapelle CA (2012). Community Interpreting. The Encyclopedia of Applied Linguistics.

[26] Pöllabauer S, Best J, Kalina S (2002). Community Interpreting als Arbeitsfeld – Vom Missionarsgeist Und Von Moralischen Dilemmata. Übersetzen Und Dolmetschen Eine Orientierungshilfe.

[27] Bancroft MA, Mikkelson H, Jourdenais R (2015). Community Interpreting. A profession rooted in social justice. The Routledge handbook of interpreting.

[28] International Standard. ISO 13611:2024. Interpreting services — Community interpreting — Requirements and recommendations. 2024;(2).

[29] Hale S (2007). Community interpreting.

[30] Pöchhacker F (2004). Introducing interpreting studies.

[31] Bancroft M, Rubio-Fitzpatrick L (2009). The Community interpreter: Professional Interpreter Training for Bilingual Staff and Community interpreters.

[32] Mikkelson H (1996). Community interpreting: an emerging profession. INTP.

[33] Hale S, Goodman-Delahunty J, Martschuk N (2019). Interpreter performance in police interviews. Differences between trained interpreters and untrained bilinguals. Interpreter Translator Train.

[34] Hlavac JA, Cross-National (2013). Overview of translator and interpreter certification procedures. Trans-Int.

[35] Hlavac J (2015). Formalizing Community Interpreting standards: a cross-national comparison of Testing systems, Certification conventions and recent ISO guidelines. Int J Interpreter Educ Int J Interpreter Educ.

[36] Martínez-Gómez A, Baker M, Saldanha G (2019). Non-professional interpreters. Routledge Encyclopedia of Translation studies.

[37] Mikkelson H (2013). Universities and interpreter certification. Trans-Int.

[38] National Accreditation Authority for Translators and Interpreters. NAATI - Annual Report 2021. Breaking down language barriers. Deakin: National Accreditation Authority for Translators and Interpreters; 2021.

[39] Iannone E (2021). Dolmetschen Im Gemeinwesen. Rahmenbedingungen Und Praxis in Deutschland. Entwicklungslinien Des Dolmetschens Im Soziokulturellen Kontext: Translationskultur(en) Im DACH-Raum.

[40] Breitsprecher C, Mueller JT, Mösko M (2020). BetweenLanguages - quality standards and minimum requirements for the qualification of interpreters in social work settings in Germany.

[41] INTERPRET (2023). Schweizerische Interessengemeinschaft für interkulturelles Dolmetschen Und Vermitteln. Einsatzstatistiken Zum Interkulturellen Dolmetschen Und Vermitteln 2022.

[42] Geiling A, Knaevelsrud C, Böttche M, Stammel N. Mental Health and Work experiences of interpreters in the Mental Health Care of refugees: a systematic review. Front Psychiatry. 2021;12(710789). 10.3389/fpsyt.2021.710789 PMC8558553 34733183

[43] Penn C, Watermeyer J (2018). Communicating across cultures and languages in the health care setting.

[44] Tiayon C (2005). Community Interpreting: an African perspective. Hermēneus Revista De Traducción E Interpretación.

[45] Geiling A, Böttche M, Knaevelsrud C, Stammel N. A comparison of interpreters’ wellbeing and work-related characteristics in the care of refugees across different work settings. BMC Public Health. 2022;22(1635). 10.1186/s12889-022-14034-7 PMC9423887 36038870

[46] Geiling A, Knaevelsrud C, Böttche M, Stammel N (2022). Psychological distress, exhaustion, and work-related correlates among interpreters working in refugee care: results of a nationwide online survey in Germany. Eur J Psychotraumatology.

[47] Kindermann D, Schmid C, Derreza-Greeven C, Huhn D, Kohl RM, Junne F (2017). Prevalence of and risk factors for secondary traumatization in interpreters for refugees: a cross-sectional study. Psychopathology.

[48] Crezee IHM, Jülich S, Hayward M (2013). Issues for interpreters and professionals working in refugee settings. JAPL.

[49] Valero Garces C (2015). The impact of emotional and psychological factors on public service interpreters: preliminary studies. Trans-Int.

[50] Grbić N, Pöllabauer S (2008). Counting what counts: Research on community interpreting in Germanspeaking countries—A scientometric study. Target.

[51] Iannone EB-D. 2020. Endlich ein differenziertes Bild. MDÜ - Fachzeitschrift für Dolmetscher und Übersetzer. 2020;(5):32–6.

[52] Pohontsch N, Meyer T (2015). Das kognitive interview – ein instrument zur Entwicklung Und Validierung Von Erhebungsinstrumenten. Rehabilitation.

[53] Mehnert A, Müller D, Lehmann C, Koch U (2006). Die deutsche version des NCCN distress-thermometers: empirische prüfung eines screening-instruments zur erfassung psychosozialer belastung bei krebspatienten. Z für Psychiatrie Psychologie und Psychother.

[54] Psychosocial Distress Practice Guidelines Panel (1999). NCCN practice guidelines for the management of psychosocial distress. Oncol (Williston Park NY).

[55] Cutillo A, O’Hea E, Person S, Lessard D, Harralson T, Boudreaux E (2017). The Distress Thermometer: cutoff points and clinical use. ONF.

[56] Riba MB, Donovan KA, Andersen B, Braun Ii, Breitbart WS, Brewer BW (2019). Distress management, Version 3.2019, NCCN Clinical Practice guidelines in Oncology. J Natl Compr Canc Netw.

[57] Ma X, Zhang J, Zhong W, Shu C, Wang F, Wen J (2014). The diagnostic role of a short screening tool—the distress thermometer: a meta-analysis. Support Care Cancer.

[58] Löwe B, Kroenke K, Gräfe K (2005). Detecting and monitoring depression with a two-item questionnaire (PHQ-2). J Psychosom Res.

[59] Hinz A, Klein AM, Brähler E, Glaesmer H, Luck T, Riedel-Heller SG (2017). Psychometric evaluation of the generalized anxiety disorder screener GAD-7, based on a large German general population sample. J Affect Disord.

[60] Löwe B, Wahl I, Rose M, Spitzer C, Glaesmer H, Wingenfeld K (2010). A 4-item measure of depression and anxiety: validation and standardization of the Patient Health Questionnaire-4 (PHQ-4) in the general population. J Affect Disord.

[61] Kroenke K, Spitzer RL, Williams JBW (2003). The Patient Health Questionnaire-2: validity of a two-item depression screener. Med Care.

[62] Cronbach LJ. Coefficient alpha and the internal structure of tests. Psychomerika. 1951;16:297–334.

[63] Tavakol M, Dennick R (2011). Making sense of Cronbach’s alpha. Int J Med Educ.

[64] Nunnally J, Bernstein L (1994). Psychometric theory.

[65] Plummer F, Manea L, Trepel D, McMillan D (2016). Screening for anxiety disorders with the GAD-7 and GAD-2: a systematic review and diagnostic metaanalysis. Gen Hosp Psychiatry.

[66] UNESCO Institute for Statistics (2012). International standard classification of education: ISCED 2011.

[67] Gentile P. The Professional Status of Public Service Interpreters. A comparison with nurses. FITISPos-International J. 2016;174–83.

[68] Bundesagentur für Arbeit. Anteil von Frauen und Männern in verschiedenen Berufsgruppen in Deutschland am 30. Juni 2022 (sozialversicherungspflichtig und geringfügig Beschäftigte). 2023.

[69] Statistisches Bundesamt (Destatis). Bevölkerung mit Migrationshintergrund - Ergebnisse des Mikrozensus 2021 (Endergebnisse) - Fachserie 1 Reihe 2.2–2021. 2023.

[70] Sommer I (2021). Recognition of foreign qualifications in Germany: selectivity and power in re-making professionals. Int Migration.

[71] Wichmann M, Nick S, Redlich A, Pawils S, Brune M, Betke E (2018). Sekundäre Traumatische ­Belastung Bei Dolmetschern in Der Flüchtlingsversorgung. Trauma Gewalt.

[72] Jurado D, Alarcón RD, Martínez-Ortega JM, Mendieta-Marichal Y, Gutiérrez-Rojas L, Gurpegui M (2017). Factors associated with psychological distress or common mental disorders in migrant populations across the world. Revista De Psiquiatría Y Salud Mental (English Edition).

[73] Mylord M, Moran JK, Özler G, Nassar R, Anwarzay S, Hintz SJ (2023). The dynamics of discrimination, resilience, and social support in the mental health of migrants with and without citizenship. Int Rev Psychiatry.

[74] Montazeri A, Harirchi AM, Shariati M, Garmaroudi G, Ebadi M, Fateh A. The 12-item General Health Questionnaire (GHQ-12): translation and validation study of the Iranian version. Health and Quality of Life Outcomes; 2003. 10.1186/1477-7525-1-66 PMC280704 14614778

[75] Norström E, Fioretos I, Gustafsson K (2012). Working conditions of community interpreters in Sweden: opportunities and shortcomings. INTP.

[76] Bundesverband der Dolmetscher und Übersetzer e.V. (BDÜ). Fiktive Aufstellung der Einnahmen/Ausgaben eines Dolmetschers im Gesundheits- und im Gemeinwesen – Beispielkalkulationen. 2022.

[77] Ozolins U (2010). Factors that determine the provision of Public Service Interpreting: comparative perspectives on government motivation and language service implementation. J Specialised Translation.

[78] Dubslaff F, Martinsen B, Brunette L, Bastin GL, Hemlin I, Clarke H (2003). Community Interpreting in Denmark: results of a survey. The critical link 3: interpreters in the community.

[79] Mikkelson H (2014). Evolution of Public Service Interpreter Training in the U.S. FITISPos-IJ.

[80] Ertl A, Pöllabauer S. Training (Medical) interpreters—the key to good practice. MedInt: a joint European training perspective. J Secialised Translation. 2014;(14):165–93.

[81] Mikkelson H. The professionalization of community interpreting. aiic.net. 2004.

[82] Ozolins U (2004). VITS Language Link: Survey of interpreting practitioners.

[83] Kruger J, Dunning D (1999). Unskilled and unaware of it: how difficulties in recognizing one’s own incompetence lead to inflated self-assessments. J Personal Soc Psychol.

[84] Wadensjö C, Baker M (2001). Community interpreting. Routledge encyclopedia of translation studies.

[85] Bahadır Ş. Das politische in stimme und blick der feldforscher_in/dolmetscher_in. In: Treiber A, Kazzazi K, Jaciuk M, editors. Migration Übersetzen. Wiesbaden: Springer Fachmedien Wiesbaden; 2020. p. 183–207.

