# Supplementary material for: Community interpreting in Germany: results of a nationwide cross-sectional study among interpreters
Source: BMC Public Health. 2024 Jun 11;24:1570. doi: 10.1186/s12889-024-18988-8 (PMC11165753; doi:10.1186/s12889-024-18988-8)
Supplement: Supplementary file 1 — Supplementary Material 1 [file 12889_2024_18988_MOESM1_ESM.docx]

**Differences between work settings**

**Age, gender and education:** Logistic regression results indicate that among the different work settings, the only settings significantly associated with older age (> 43 years, median) are authorities (OR: 1.66, CI 95%: 1.10 - 2.49, p-value = 0.02) and court (OR: 2.16, CI 95%: 1.47 – 3.19, p-value < 0.00). The legal setting was significantly associated with male gender (OR: 1.97, CI 95%: 1.38 – 2.83, p-value < 0.00). Healthcare (OR: 0.28, CI 95%: 0.08 – 0.76, p-value = 0.02), psychotherapy (OR: 2.37, CI 95%: 1.11 – 5.09, p-value = 0.03) and authorities (OR: 2.8, CI 95%: 1.07 – 7.33, p-value = 0.04) were significantly associated with higher education.

**Interpreting experience:** In terms of interpreting experience, only court and police are significantly associated with more work experience (> 6 years, median) (OR: 3.73, CI 95%: 2.24 – 6.21, p-value < 0.00 and OR: 2.51, CI 95%: 1.59– 3.97, p-value < 0.00).

**Frequency of interpreting:** Only healthcare (OR: 1.71, CI 95%: 1.23 – 2.39, p-value < 0.00), legal settings (OR: 2.61, CI 95%: 1.8 – 3.77, p-value < 0.00) and police (OR: 1.57, CI 95%: 1.07 – 2.31, p-value = 0.02) are significantly associated with working more frequently as CIP (> 10 hours per month, median).

**Qualification and interpreting competence:** Logistic regression results showed that only the settings of psychotherapy (OR: 2.29, CI 95%: 1.59 – 3.29, p-value < 0.00), education (OR: 1.59, CI 95%: 1.12 – 2.26, p-value = 0.01), legal (OR: 1.69, CI 95%: 1.1 – 2.6, p-value = 0.02) and court (OR: 1.83, CI 95%: 1.1 – 3.07, p-value = 0.02) are significantly associated with CIPs being somehow trained. Similarly, these settings are significantly associated with CIPs having received more than 25 hours of training (median) (OR: 1.47, CI 95%: 1.02 – 2.11, p-value = 0.04; OR: 2.03, CI 95%: 1.39 – 2.97, p-value < 0.00; OR: 2.13, CI 95%: 1.41 – 3.22, p-value < 0.00). People working in healthcare (OR: 1.99, CI 95%: 1.21 – 3.28, p-value = 0.01), at authorities (OR: 0.57, CI 95%: 0.33 – 0.97, p-value = 0.04), in legal settings (OR: 2.36, CI 95%: 1.53 – 3.64, p-value < 0.00) or in court (OR: 2.33, CI 95%: 1.49 – 3.64, p-value < 0.00) are significantly more likely to have completed an interpreting exam. Regarding subjective interpreting competence, only people working in psychotherapy (OR: 1.97, CI 95%: 1.33 – 2.91, p-value < 0.00) or police settings (OR: 2.05, CI 95%: 1.14 – 3.7, p-value = 0.02) are significantly more likely to perceive themselves as rather or very competent.

**Frequency and amount of payment:** Only the settings of psychotherapy (OR: 1.98, CI 95%: 1.17 – 3.37, p-value = 0.01), education (OR: 1.79, CI 95%: 1.1 – 2.91, p-value = 0.02) or court (OR: 2.85, CI 95%: 1.27 – 6.37, p-value = 0.01) are significantly associated with CIPs always getting paid. Additionally, people working in psychotherapy (OR: 3.09, CI 95%: 2.15 – 4.44, p-value < 0.00), social services (OR: 0.63, CI 95%: 0.42 – 0.95, p-value = 0.03), authorities (OR: 0.63, CI 95%: 0.4 – 1.0, p-value = 0.05), legal settings (OR: 1.89, CI 95%: 1.26 – 32.82, p-value < 0.00) and court (OR: 6.09, CI 95%: 3.68 – 10.06, p-value < 0.00) are significantly more likely to receive payment above 20 Euros (median).
