# Supplementary material for: Community interpreting in Germany: results of a nationwide cross-sectional study among interpreters
Source: BMC Public Health. 2024 Jun 11;24:1570. doi: 10.1186/s12889-024-18988-8 (PMC11165753; doi:10.1186/s12889-024-18988-8)
Supplement: Supplementary file 2 — Supplementary Material 2 [file 12889_2024_18988_MOESM2_ESM.docx]

| **Table 2.1** Availability of interpreting-related support services (N = 873). | | |
| --- | --- | --- |
|  | **n** | **%** |
| **Contact person for organizational issues related to interpreting** | **746** |  |
| Yes | 541 | 72.5 |
| I do not know | 51 | 6.8 |
| No | 154 | 20.6 |
| I do not know/No, but I would like to have it | 110 | 53.9 |
| I do not know/No, and I would not like to have it | 48 | 23.5 |
| I do not know/No, and I do not know if I would like to have it | 46 | 22.6 |
| *Missing value* | *127* | - |
| **Contact person for content-related issues** | **746** |  |
| Yes | 332 | 44.5 |
| I do not know | 82 | 11 |
| No | 332 | 44.5 |
| I do not know/No, but I would like to have it | 222 | 53.8 |
| I do not know/No, and I would not like to have it | 118 | 28.6 |
| I do not know/No, and I do not know if I would like to have it | 73 | 17.7 |
| *Missing value* | *127* | - |
| **Supervision** | **745** |  |
| Yes | 324 | 43.5 |
| I do not know | 143 | 19.2 |
| No | 278 | 37.3 |
| I do not know/No, but I would like to have it | 185 | 43.9 |
| I do not know/No, and I would not like to have it | 112 | 26.6 |
| I do not know/No, and I do not know if I would like to have it | 124 | 29.5 |
| *Missing value* | *128* | - |
| **Intervision** | **745** |  |
| Yes | 386 | 51.8 |
| I do not know | 82 | 11.0 |
| No | 277 | 37.2 |
| I do not know/No, but I would like to have it | 181 | 50.4 |
| I do not know/No, and I would not like to have it | 86 | 24 |
| I do not know/No, and I do not know if I would like to have it | 92 | 25.6 |
| *Missing value* | *128* | - |
| **Interpreting-specific training** | **745** |  |
| Yes | 557 | 74.8 |
| I do not know | 42 | 5.6 |
| No | 146 | 19.6 |
| I do not know/No, but I would like to have it | 132 | 69.8 |
| I do not know/No, and I would not like to have it | 29 | 15.3 |
| I do not know/No, and I do not know if I would like to have it | 28 | 14.8 |
| *Missing value* | *128* | - |
